# Cold Atmospheric Plasma in Periodontitis: Mechanisms, Antimicrobial and Immunomodulatory Effects, and Therapeutic Potential—A Comprehensive Review

**DOI:** 10.3390/dj14080468

**Published:** 2026-08-02

**Authors:** Abbas Bumajdad, Ali Alnaser, Mohammad Qali

**Affiliations:** 1 Ministry of Health, Kuwait City 13001, Kuwait; bumajdad1@gmail.com (A.B.); dr.ali.alnaser@outlook.com (A.A.); 2 Department of Surgical Sciences, College of Dentistry, Health Sciences Center, Kuwait University, Kuwait City 13060, Kuwait

**Keywords:** cold atmospheric plasma, periodontitis, antimicrobial effects, biofilms, immunomodulation, periodontal regeneration

## Abstract

**Background:** Periodontitis is a chronic inflammatory disease characterized by a dysbiotic oral microbiome that leads to progressive periodontal tissue destruction. Current treatment primarily relies on scaling and root planing (SRP); however, this approach does not always completely eliminate pathogenic microorganisms or adequately modulate the host inflammatory response. Cold atmospheric plasma (CAP) generates reactive oxygen and nitrogen species (RONS) with potential antimicrobial, antibiofilm, and immunomodulatory properties, making it a promising adjunctive therapeutic modality for the management of periodontitis. **Literature Search Strategy:** A comprehensive literature search was conducted using PubMed/MEDLINE, Scopus, Web of Science, and Embase to identify relevant studies published between January 2000 and June 2025. The search included in vitro, in vivo, and clinical studies evaluating the antimicrobial, immunomodulatory, and regenerative effects of CAP in periodontitis and related periodontal models. Reference lists of relevant articles were also manually screened to identify additional eligible studies. **Objectives:** This review aims to provide a comprehensive overview of the antimicrobial and immunomodulatory effects of CAP on periodontitis and to evaluate its potential as an adjunctive treatment modality. Furthermore, the review examines the regenerative potential and underlying mechanisms of CAP, while also addressing its clinical safety and biocompatibility. **Key Findings:** Preclinical evidence indicates that CAP reduces bacterial viability, disrupts biofilm architecture, and modulates host immune responses through RONS-mediated signaling pathways. In animal models of periodontitis, CAP has been shown to enhance markers of periodontal tissue repair, reduce the expression of pro-inflammatory cytokines, increase LC3-associated phagocytosis in macrophages, and accelerate wound healing. Clinical studies evaluating CAP as an adjunct to SRP have reported improvements in clinical attachment level (CAL) and reductions in the recolonization of periodontal pathogens. Nevertheless, substantial heterogeneity in CAP treatment parameters and a lack of long-term safety and efficacy data limit direct comparisons across studies and highlight the need for further well-designed clinical investigations. **Conclusions:** CAP appears to be a potentially useful adjunct to SRP in the management of periodontitis. Current preclinical evidence demonstrates significant antimicrobial, immunomodulatory, and regenerative effects; however, clinical evidence remains limited. Further high-quality clinical trials are required to validate these findings, establish standardized treatment protocols, optimize therapeutic parameters, and facilitate the development of regulatory frameworks for clinical implementation.

## 1. Introduction

Periodontitis is a chronic inflammatory disease caused by dysbiotic microbial biofilms and dysregulated host immune responses, leading to periodontal tissue destruction characterized by alveolar bone loss and clinical attachment loss [1]. It is a widespread disease and a major contributor to tooth loss in adults [1]. A keystone pathogen involved in disease pathogenesis is *Porphyromonas gingivalis*, alongside other pathobionts, including *Treponema denticola* and *Tannerella forsythia*. Collectively, these organisms constitute the “red complex” bacteria and are considered major etiological agents of periodontal disease [1]. The pathophysiology of periodontitis involves complex interactions between red complex bacteria and host immunoinflammatory responses. These interactions are mediated by bacterial virulence factors, such as lipopolysaccharides (LPS) and proteases, which activate the innate immune system and promote the release of pro-inflammatory cytokines (e.g., IL-1β, IL-6, and TNF-α), chemokines (e.g., IL-8), prostaglandins (PGs), and matrix metalloproteinases (MMPs) [1]. This exaggerated inflammatory response ultimately results in periodontal tissue destruction, alveolar bone loss, and potential tooth loss [1].

SRP, with or without adjunctive antimicrobial agents, represents the main therapeutic approach for the management of periodontitis. The European Federation of Periodontology (EFP) S3-level clinical practice guidelines recommend a stepwise approach for the management of periodontitis, with Step 2 focusing on subgingival instrumentation aimed at disrupting and removing subgingival biofilm and calculus deposits [2]. In selected clinical situations, adjunctive therapies such as locally delivered antimicrobials may be considered to enhance treatment outcomes [2]. Although SRP with or without adjunctive local antimicrobial therapy usually results in clinical improvement, many limitations exist, such as persistent inflammation and incomplete resolution of host-mediated tissue destruction [3]. Furthermore, bacterial recolonization, biofilm resilience, and antibiotic resistance present additional therapeutic challenges [3]. These limitations highlight the need for adjunctive interventions that can modulate host inflammatory and immune responses while simultaneously suppressing pathogenic mechanisms of dental biofilms. CAP has been proposed as a strategy to reduce the microbial burden within periodontal pockets. CAP achieves this effect through the generation of reactive oxygen and nitrogen species that exert antimicrobial, antibiofilm, and immunomodulatory actions [4]. Consequently, CAP represents a promising emerging adjunctive modality that may complement conventional non-surgical periodontal therapy and warrants further investigation in the management of periodontitis [4].

CAP, also known as nonthermal atmospheric pressure plasma (NAPP), is a partially ionized gas composed of reactive components such as free electrons, positively charged ions, neutral particles, reactive radicals, and photons including ultraviolet (UV) radiation [5,6]. It is generated by applying high voltage to a gas at atmospheric pressure to induce ionization, using methods such as pulse discharges, microwave discharges, and dielectric barrier discharge (DBD) systems with electrodes separated by a dielectric material [6]. When sufficient energy is applied, collisions between electrons and neutral gas atoms result in ionization and CAP formation.

The chemical and physical properties of CAP underlie its biological effects. Chemically, CAP is highly reactive due to reactive species and free electrons that can induce oxidative stress and disrupt pathogen cellular components [7,8], and it is electrically conductive because of its charged particles. Physically, CAP operates at room temperature and atmospheric pressure, making it accessible and suitable for clinical use. These properties contribute to a wide range of biological and clinical effects in dentistry, including antimicrobial activity, immunomodulation, tissue remodeling, and wound healing. Because CAP is nonthermal, energy is carried by high-energy electrons while the gas remains near room temperature, allowing direct interaction with periodontal tissues without thermal damage [6]. Its chemical and physical characteristics enable selective targeting of pathogens with minimal harm to healthy tissues, supporting its clinical use in dentistry.

**Figure 1:**
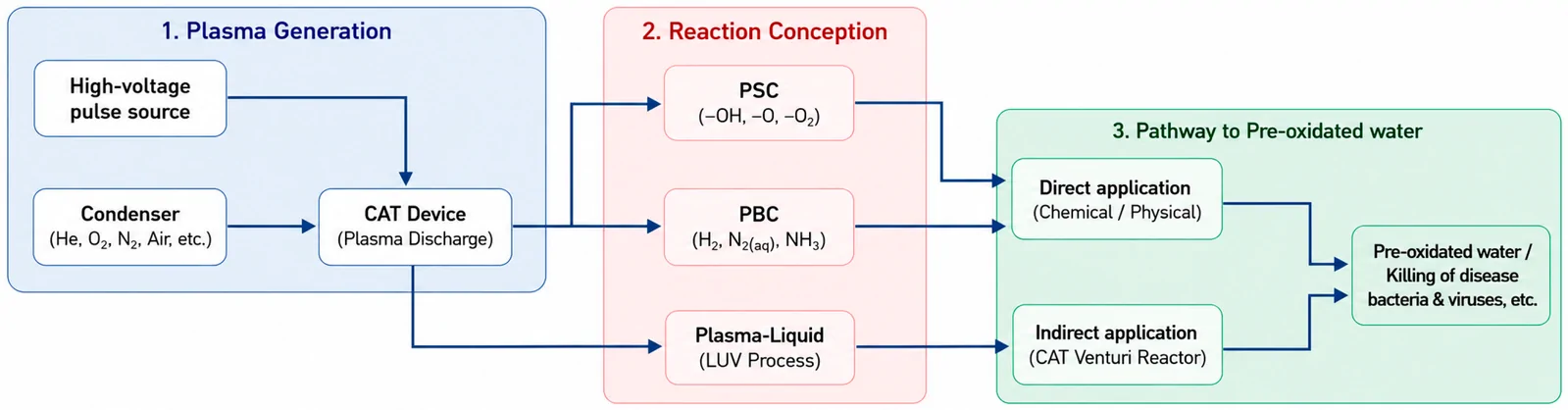
Formation of Cold Atmospheric Plasma and Delivery in Periodontal Therapy. High-voltage power supplied to a CAP device, together with a carrier gas (He, Ar, Air, or N_2_), generates reactive oxygen species (ROS), reactive nitrogen species (RNS), electric fields, and UV photons. These bioactive components can be delivered directly to periodontal pockets via a plasma jet or indirectly through CAP-treated liquids. The generated reactive oxygen and nitrogen species (RONS) interact with the periodontal biofilm and surrounding tissues, exerting antimicrobial and immunomodulatory effects while minimizing thermal damage to host tissues.

The composition and activity of CAP depend on the type of gas used (e.g., helium, argon, or nitrogen) and the plasma production system [9], while its efficiency is influenced by factors such as gas flow rate, power frequency, and chamber geometry [8]. Clinically, CAP may be applied directly to target tissues or indirectly through CAP-treated liquids delivered to the treatment site [10,11]. CAP generation, composition, and delivery are summarized in Figure 1.

The aim of this review is to provide an overview of the current evidence on CAP’s antimicrobial and immunomodulatory effects on periodontitis with emphasis on the underlying molecular mechanisms. It further evaluates the effects of CAP on periodontal tissue healing and regeneration and summarizes findings from in vivo and in vitro studies to evaluate its efficacy in the management of periodontitis. In addition, this review assesses periodontal treatment outcomes when CAP is used as an adjunct to conventional therapy and compares it with alternative periodontal adjuncts. Furthermore, the clinical safety and biocompatibility of CAP will be discussed. Finally, the review highlights research gaps and provides guidance on future research pathways.

## 2. Methods and Literature Search Strategy

### 2.1. Methods

This comprehensive literature review was conducted to synthesize current evidence on the antimicrobial, immunomodulatory, and tissue-supportive effects of cold CAP in the context of periodontitis, with particular emphasis on biological mechanisms, translational relevance, and safety and biocompatibility profiles. The review aimed to integrate mechanistic, preclinical, and clinical evidence rather than to perform quantitative effect estimation or formal evidence grading.

### 2.2. Literature Search Strategy

This comprehensive review synthesizes current evidence on the application of CAP in periodontal therapy, with a focus on antimicrobial mechanisms, immunomodulatory effects, therapeutic efficacy, and safety considerations. A structured literature search was conducted using major biomedical databases, including PubMed/MEDLINE, Scopus, and Web of Science, to identify relevant experimental, preclinical, and translational studies. The literature search covered studies published between 2000 and 2025 to capture both the foundational investigations that established the biological and antimicrobial principles of CAP and the most recent studies evaluating its applications in periodontal therapy. This broader timeframe was selected to provide a comprehensive overview of the evolution of CAP research and its potential role in periodontitis management. Studies of different designs, including in vitro investigations, animal studies, and clinical trials, were included to provide a comprehensive evaluation of the current evidence regarding the antimicrobial and immunomodulatory effects of CAP in periodontitis. Given the emerging nature of this field and the limited availability of clinical data, inclusion of multiple study types enabled the synthesis of mechanistic, preclinical, and clinical evidence, thereby offering a broader understanding of the therapeutic potential and biological effects of CAP. Studies were excluded if they lacked relevant outcome measures, were conference abstracts, editorials, letters to the editor, expert opinions, duplicate publications, non-English abstracts, or were not available in full-text format. Following the screening and eligibility assessment process, a total of 106 studies met the inclusion criteria and were included in this review.

Search terms were selected to capture the breadth of CAP-related periodontal research and included combinations of keywords relating to cold atmospheric plasma, non-thermal plasma, periodontitis, periodontal disease, biofilm, reactive oxygen and nitrogen species, inflammation, immunomodulation, osteoclastogenesis, periodontal regeneration, safety, and biocompatibility. Reference lists of eligible articles were additionally screened to identify further relevant studies.

Given the heterogeneity of plasma devices, treatment parameters, and outcome measures across the available literature, a narrative synthesis approach was adopted. This approach enabled integration of mechanistic and translational findings that could not be readily compared through quantitative meta-analysis. Studies were evaluated for relevance to periodontal disease pathogenesis, biological plausibility, and potential clinical applicability.

## 3. Results: Molecular Mechanisms of CAP’s Antimicrobial and Immunomodulatory Actions

The following section summarizes CAP’s antimicrobial, anti-biofilm, and immunomodulatory effects with an emphasis on CAP’s molecular mechanisms. A brief summary on the formation of CAP and its delivery in periodontal therapy is shown in Figure 1.

### 3.1. CAP’s Antimicrobial and Antibiofilm Mechanisms

**Figure 2:**
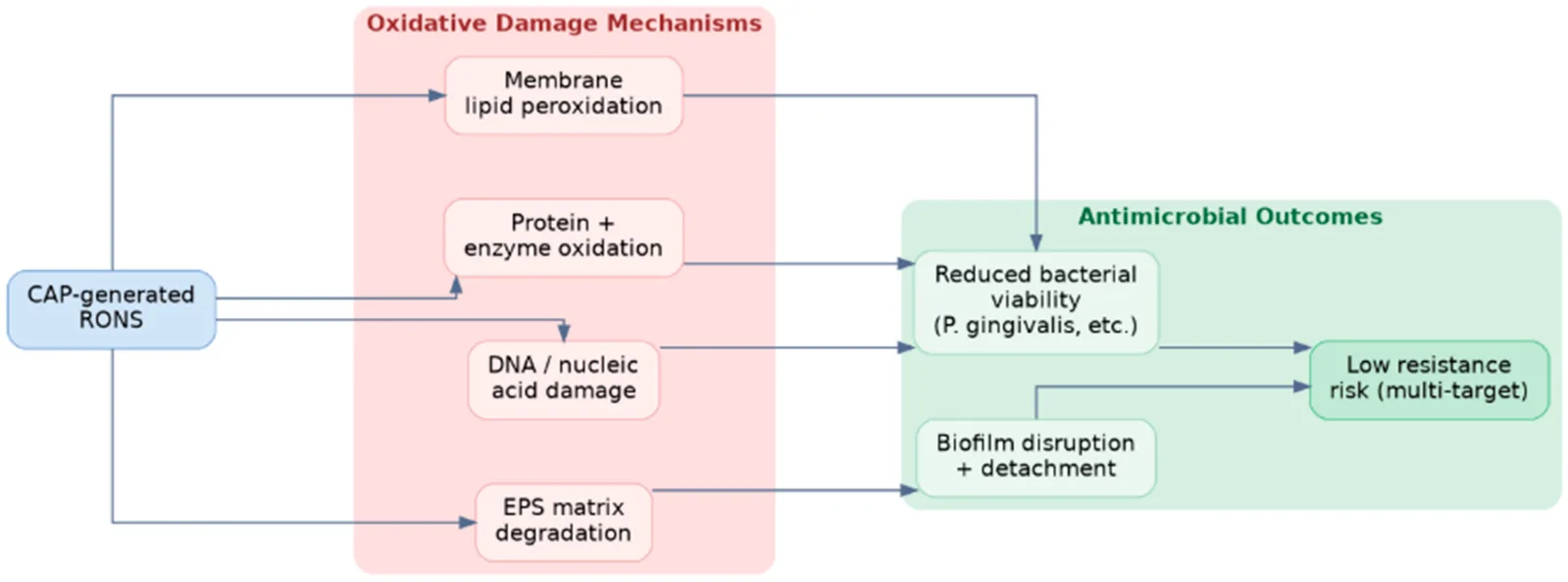
Proposed Antimicrobial and Antibiofilm Mechanisms of Cold Atmospheric Plasma (CAP). CAP-generated RONS induce oxidative damage through multiple pathways, including membrane lipid peroxidation, protein and enzyme oxidation, DNA and nucleic acid damage, and degradation of the extracellular polymeric substance (EPS) matrix. These effects reduce bacterial viability and disrupt biofilm structure, resulting in biofilm detachment and enhanced microbial inactivation. The simultaneous targeting of multiple cellular and extracellular components contributes to the broad-spectrum antimicrobial activity of CAP and may reduce the likelihood of resistance development.

The inhibitory mechanisms of CAP have been characterized based on its interactions with specific bacterial structural and functional components. These mechanisms include damage to the cell wall and cytoplasmic membrane, oxidative stress and intracellular damage, degradation and post-translational modification of proteins, chemical modification and strand breakage of nucleic acids, as well as disruption of extracellular polymeric substances (EPS) and interference with quorum sensing (QS) pathways within biofilms [8,12,13]. A summary of CAP’s proposed antimicrobial and antibiofilm mechanisms is shown in Figure 2. This subsection focuses on the destructive effects of CAP on distinct bacterial components and their contribution to antimicrobial activity as well as CAP’s effects on biofilms.

Production of reactive species

CAP generates reactive species including hydrogen peroxide (H_2_O_2_), hydroxyl radicals (•OH), ozone (O_3_), and nitric oxide (NO) through radical recombination, electron-impact dissociation, and gas-phase chemistry [8]. Its antimicrobial mechanisms are primarily due to the production of RONS, with each species showing distinct redox reactivity and selective biological targets [13]. The biological effects of CAP depend on plasma operating conditions and reactive species composition [14]. Air-fed CAP predominantly generates ROS that act at cell surfaces, whereas nitrogen-fed CAP generates higher levels of RNS that penetrate cells and induce intracellular damage, producing distinct transcriptomic responses related to stress and metabolic pathways [14].

Operational parameters such as delivered power, voltage, humidity, and exposure time influence free radical production and accumulation of long-lived species like hydrogen peroxide, modulating the selectivity and potency of CAP-induced bactericidal effects [15,16]. The ability to tune RONS generation allows CAP to be tailored for maximum antimicrobial efficacy [15,16]. Synergism among reactive species enhances antimicrobial activity, with short-lived radicals causing immediate surface oxidation and longer-lived species providing sustained activity and deeper biofilm penetration [13,17]. Understanding the spatial distribution and temporal dynamics of reactive species is essential for optimizing CAP use in PD.

Bacterial cell wall and membrane damage

Highly virulent periodontal pathogens are predominantly Gram-negative anaerobes [1]. However, Gram-positive periodontal species (i.e., *Streptococcus sanguinis*, *Streptococcus gordonii*, and *Actinomyces naeslundii*) are important because they form the initial biofilm scaffold that enables adhesion of Gram-negative periodontopathogens [18,19]. Gram-positive bacteria are also used as reference organisms in CAP studies due to their thick peptidoglycan walls and lack of LPS and an outer membrane [9,20]. Comparing Gram-positive and Gram-negative responses helps clarify CAP selectivity and optimize plasma properties such as gas type [21]. Additionally, Gram-positive bacteria occupy outer biofilm layers and partially protect deeper Gram-negative anaerobes, so CAP effects on them can improve RONS penetration and antibacterial access to subgingival pathogens [12,22].

CAP’s antimicrobial action is based on interactions between CAP-generated RONS and bacterial envelopes, with variable effects due to structural differences [13,20]. Gram-negative bacteria show rapid membrane oxidation and higher permeability due to LPS-rich outer membranes, while Gram-positive bacteria possess thicker peptidoglycan layers that provide some resilience but remain susceptible to RONS-mediated oxidation [9,13,20]. CAP-generated RONS can cause phospholipid peroxidation, membrane depolarization, leakage of intracellular contents, and structural damage to peptidoglycans and outer membranes, enhancing RONS diffusion and disrupting cellular homeostasis [23,24]. These effects lead to membrane disruption, degradation of motility structures, and damage to virulence factors and adhesins, contributing to bactericidal activity and reduced pathogenicity [9,12,14].

Oxidative stress and intracellular damage

Following disruption of the bacterial envelope, reactive species generated by CAP enter the cytosol, where they exhaust antioxidant machinery and dysregulate essential cellular pathways [9,13]. CAP exposure induces protein oxidation and transcriptional reprogramming consistent with RONS-driven stress responses [9]. Bacterial redox systems such as catalase, glutathione-mediated enzymes, and superoxide dismutase maintain balance under physiological conditions but exceed their buffering capacity during CAP-induced oxidative stress, leading to non-repairable injury once a critical threshold is surpassed [9,13].

ROS-predominant exposures trigger metabolic downregulation and efflux activity, whereas RNS-dominated exposures cause more extensive intracellular damage, including disruption of ribosomal function and biosynthetic processes, reflecting distinct intracellular injury patterns [14]. These differences relate to the higher membrane penetration and longer lifetime of RNS [13,14]. Although transcriptomic responses show adaptive mechanisms such as DNA repair and metabolic reprogramming, these are overwhelmed with prolonged CAP exposure [14]. Subsequent oxidative damage includes thiol oxidation, loss of redox enzyme activity, metabolic collapse, and disruption of replication and repair pathways, ultimately compromising cell viability [14,23]. Accumulation of secondary oxidants further damages proteins, nucleic acids, and lipids, impairing biosynthesis, energy metabolism, and genome maintenance and preventing recovery [13,25].

CAP effects on bacterial DNA and proteins

CAP exposure induces oxidative damage to bacterial DNA and proteins, impairing replication and enzymatic activity [13,24]. Reported lesions include DNA strand breaks, base alterations, oxidative adducts, and protein carbonylation that disrupt folding and function [13]. Mechanisms include nucleobase oxidation, especially guanine, deoxyribose backbone oxidation causing strand breaks, and DNA–protein crosslinks that impede replication and transcription, producing lesions such as single and double strand breaks, abasic sites, and 8-oxo-guanine that overwhelm repair systems [12,13,23].

RNS-rich CAP disrupts ribosome-associated gene expression and nucleic acid-processing pathways, reducing translational capacity and impairing cellular maintenance and repair [9,14]. Oxidative protein modification causes enzyme inactivation and metabolic dysfunction, while extensive DNA damage inhibits replication, producing bacteriostatic and bactericidal effects [13,24]. These multi-target insults limit classical resistance development because simultaneous damage to membranes, proteins, and nucleic acids imposes a high adaptive barrier [13,20]. Unlike conventional antibiotics that act on discrete targets, CAP compromises multiple cellular systems, providing an advantage against antimicrobial resistance [13].

CAP effects on apoptosis-like cell death in bacteria

Bacterial exposure to CAP can produce apoptosis-like features such as membrane depolarization, DNA fragmentation, and morphological changes, reflecting regulated bacterial cell death with similarities to eukaryotic apoptosis [23,26,27]. Although bacteria lack canonical apoptotic machinery, CAP-induced oxidative stress can activate nucleases, proteases, and autolytic pathways, leading to coordinated cellular disintegration beyond direct chemical damage [23,28]. The balance between necrotic-like lysis and apoptosis-like responses depends on CAP dose and reactive species composition, with RNS-enriched conditions promoting intracellular damage and apoptosis-associated features [23,28].

These responses have therapeutic implications. Sublethal CAP doses that engage regulated death pathways may achieve antimicrobial effects while limiting release of pro-inflammatory contents from necrotic lysis [13,23,28]. Activation of regulated death mechanisms may also reduce persistence of viable but non-culturable subpopulations that contribute to disease recurrence [13,23,28]. Understanding CAP-induced death pathways supports optimization of treatment protocols to maximize microbial eradication while minimizing undesirable biological effects.

CAP’s effects on biofilms:

Biofilms are structured microbial communities adherent to a tissue matrix rich in EPS composed of phospholipids, proteins, extracellular DNA, and polysaccharides, providing protection, communication, and adaptability that enhance survival under stress [24,29]. These characteristics make biofilms difficult to eliminate without promoting resistance [13,23,25].

Studies included in this review demonstrated that CAP exhibits antimicrobial activity against several periodontal pathogens implicated in the initiation and progression of periodontitis, including *Porphyromonas gingivalis*, *Aggregatibacter actinomycetemcomitans*, *Fusobacterium nucleatum*, *Prevotella intermedia*, and *Tannerella forsythia* [2,11]. In addition to its effects on individual bacterial species, CAP has shown efficacy against complex multispecies periodontal biofilms that more closely resemble the microbial communities present within periodontal pockets [2].

CAP exerts antimicrobial effects on biofilms similar to planktonic bacteria, including damage to membranes, proteins, and nucleic acids, but also disrupts EPS and modulates quorum sensing (QS) pathways [12,30,31]. QS is a population-dependent communication system regulated by autoinducers that coordinate biofilm development, virulence, and antimicrobial tolerance [32]. CAP can interfere with QS by degrading signaling molecules such as acyl-homoserine lactones and downregulating QS-associated genes, reducing signal availability and attenuating virulence and biofilm pathogenicity [33,34].

The EPS matrix promotes cell proximity, communication, and gene transfer within biofilms [35]. CAP-generated RONS disrupt EPS structural integrity, reducing cohesion, surface adhesion, and stability, leading to mechanical destabilization and biofilm detachment [35,36]. By impairing EPS and QS pathways, CAP enhances biofilm dispersal and increases susceptibility of biofilm-associated bacteria to inactivation, demonstrating strong antibiofilm activity [33,34,35,36].

### 3.2. Additional Antimicrobial Mechanisms of CAP

The antimicrobial effects of CAP are primarily due to RONS, but other components such as UV, temperature, pH, electric fields, and charged particles also contribute to bactericidal activity [13].

Mechanism of UV on bacteria

UV radiation includes UVA, UVB, and UVC, with UVC showing the highest bactericidal efficacy through DNA damage such as thymine dimer formation, along with protein oxidation and membrane destabilization [37,38,39,40,41]. However, UV is not the principal antimicrobial mechanism of CAP because CAP-generated UV intensity is low and consists mainly of UVA and UVB with weaker direct antimicrobial activity [42,43].

Mechanism of temperature on bacteria

Although CAP can cause slight localized heating, the temperature rise is minimal and does not account for primary antibacterial effects, despite potentially promoting ROS generation [13,44,45].

Mechanism of pH on bacteria

CAP can alter pH through nitrogen oxide-derived acids or, in some conditions, increase pH via electrochemical reactions [13,46]. While pH changes alone have limited bactericidal effects, they enhance antimicrobial efficacy by increasing membrane permeability and reactive species activity [13,47,48,49,50,51].

Electric fields and charged particles

CAP-generated electric fields and charged particles can disrupt membranes via electrostatic stress and electroporation, contributing to bacterial inactivation but not fully explaining CAP’s overall efficacy [9,13,52,53,54,55,56,57].

### 3.3. CAP’s Immunomodulatory Mechanisms

**Figure 3:**
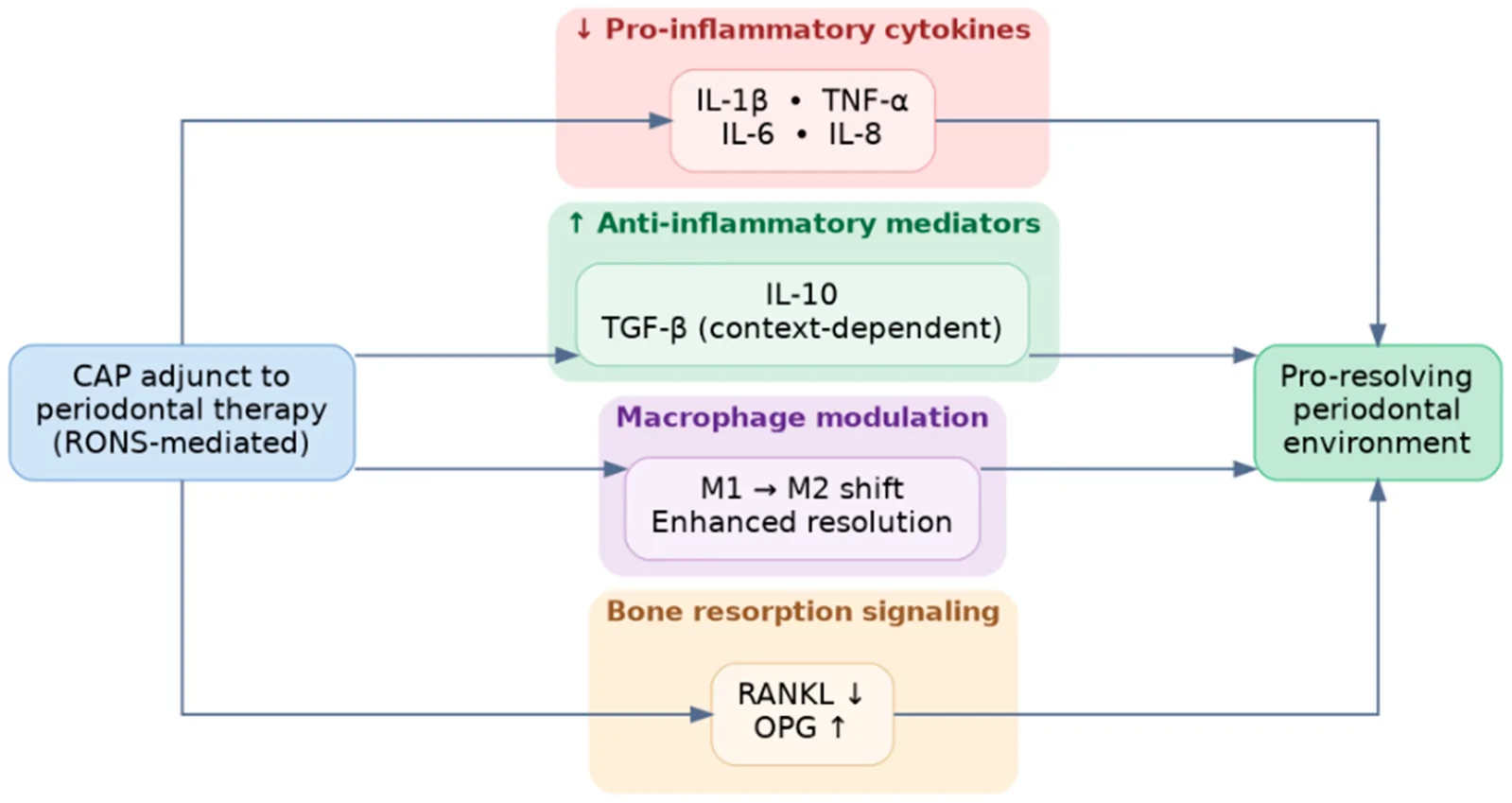
Proposed Immunomodulatory Mechanisms and Effects of Cold Atmospheric Plasma. CAP, primarily through the actions of reactive oxygen and nitrogen species (RONS), modulates the host inflammatory response by reducing the expression of pro-inflammatory cytokines, including interleukin (IL)-1β, IL-6, IL-8, and tumor necrosis factor-alpha (TNF-α), while promoting anti-inflammatory mediators such as IL-10 and, in certain contexts, transforming growth factor-beta (TGF-β). CAP may also influence macrophage polarization by promoting a shift from the pro-inflammatory M1 phenotype toward the pro-resolving M2 phenotype, thereby enhancing the resolution of inflammation. In addition, CAP has been associated with modulation of bone remodeling pathways through decreased receptor activator of nuclear factor kappa-B ligand (RANKL) expression and increased osteoprotegerin (OPG) expression. Collectively, these effects contribute to the establishment of a pro-resolving periodontal environment that may support periodontal tissue healing and regeneration.

CAP’s immunomodulatory effects occur through various interlinked mechanisms, most notably through the RONS pathway which regulates cellular signaling cascades, downregulates pro-inflammatory cytokine production, and modulates gene expression in periodontal cells and tissues [58,59,60,61]. Through these pathways, CAP can result in a reduction in inflammation, reduction in periodontopathogen burden, and improvements in periodontal tissue healing and regeneration [58,60,61,62]. A summary of CAP’s proposed immunomodulatory mechanisms and effects are presented in Figure 3, with key cytokines summarised in Appendix A.

Periodontal cytokines:

Periodontitis is an inflammatory condition characterized by a dysregulated cytokine network caused by microbial biofilms and overactivation of the immune system [1]. Cytokines play a pivotal role in the inflammatory cascade of periodontitis by directing immune cell recruitment and activation, regulating tissue degradation and repair, and determining disease outcomes [1]. The primary goal of CAP in periodontitis is to rebalance the dysregulated cytokine milieu [61]. Therefore, understanding CAP-mediated modulation of the periodontal cytokine milieu is fundamental to elucidating its therapeutic potential.

CAP effects on pro-inflammatory cytokines:

CAP modulates the expression of pro-inflammatory cytokines in periodontal tissues and gingival crevicular fluid (GCF), particularly IL-1β, IL-6, and TNF-α, which are mainly produced by gingival fibroblasts, epithelial cells, and macrophages in periodontitis [63,64,65]. IL-1β is a potent mediator of tissue destruction through MMP activation, osteoclast stimulation, and amplification of inflammation, and CAP use is associated with reduced IL-1β levels in periodontal tissues [63,64].

IL-6 contributes to acute-phase responses, B-cell differentiation, and alveolar bone resorption, and emerging evidence shows CAP treatment can reduce IL-6 expression and interrupt inflammatory signaling involved in tissue destruction [9,58,64,66]. Studies demonstrate that non-invasive physical plasma reduces IL-1β and IL-6 in pathogen-stimulated gingival fibroblasts and suppresses inflammatory proteins and cytokines in *P. gingivalis* LPS-stimulated cells, with repeated treatment reducing inflammatory infiltration and osteoclast activity in periodontitis models [62,67].

TNF-α is a key regulator of periodontal inflammation and bone loss, and experimental studies indicate CAP can modulate TNF-α expression, leading to reduced inflammatory cell recruitment, decreased inflammatory mediators, and attenuation of bone resorption [56,60,61,62,63,64].

CAP effects on chemokines and immune cell recruitment:

IL-8 is an important chemokine that plays a key role in periodontal inflammation. It acts as a potent neutrophil chemoattractant and promotes neutrophil recruitment and activation, resulting in connective tissue degradation and alveolar bone loss through chemokine and protease-mediated pathways [64,68]. Eggers et al. showed that exposure of human gingival fibroblasts (HGFs) to *Fusobacterium nucleatum* induces pronounced upregulation of IL-6 and IL-8, which is effectively attenuated by NIPP treatment [67]. Suppression of IL-8 expression suggests a potential role for CAP in regulating leukocyte recruitment, particularly neutrophil influx, thereby reducing inflammation-driven periodontal tissue damage.

CAP effects on anti-inflammatory cytokines:

CAP can upregulate and enhance the effects of anti-inflammatory cytokines, such as IL-10 and TGF-β, which are involved in the resolution of inflammation and macrophage polarization toward a pro-healing (M2) phenotype, thereby supporting tissue repair processes [7,54,57]. By shifting the cytokine balance toward an anti-inflammatory profile, CAP may contribute to the preservation of alveolar bone and the promotion of periodontal tissue healing [58,63]. Although direct mechanistic data on the modulation of TGF-β by CAP in periodontitis is sparse, the documented regenerative effects of CAP indicate potential positive modulatory influence on TGF-β- signaling pathways within periodontal tissues [58,63].

RONS effects:

CAP exerts immunomodulatory and regenerative effects through RONS that act as secondary messengers influencing intracellular signaling without causing thermal damage to periodontal tissues [9,58,59,60]. Helium CAP has been shown to reduce mitochondrial and cytoplasmic ROS levels, producing a hormetic response linked to enhanced antioxidant defenses such as glutathione peroxidase 4 upregulation, and may involve redox-sensitive pathways including Nrf2 [58,59,61]. CAP also decreases mRNA expression of inducible nitric oxide synthase and cyclooxygenase-2, indicating modulation of key inflammatory enzymes [58,59,61].

Plasma-activated medium (PAM), which contains long-lived RONS, has demonstrated immunomodulatory and autophagy-related effects associated with periodontal regeneration and reduced pro-inflammatory macrophage markers [13,42,69]. Long-lived species such as hydrogen peroxide, nitrite, and nitrate provide sustained biological activity, and their combination with short-lived RONS may prolong therapeutic effects [13].

Controlled CAP-derived RONS exposure can attenuate pathogen-induced upregulation of IL-6 and IL-8 in periodontal ligament cells without significant cytotoxicity at therapeutic doses, highlighting the importance of optimizing treatment parameters to achieve effective RONS levels while maintaining cellular viability [70].

### 3.4. CAP Effects on the Immune System

Macrophage function and phagocytosis

CAP can improve immune clearance of *P. gingivalis* through LC3-associated phagocytosis (LAP) in macrophages [71]. CAP promotes microtubule-associated protein 1A/1B-light chain 3-associated phagocytosis, enhancing intracellular destruction of *P. gingivalis* and demonstrating an innate immune response that bridges autophagy and phagocytosis [71,72]. Unlike canonical autophagy, LAP selectively targets pathogen-containing phagosomes, enabling more efficient intracellular bacterial degradation and helping explain enhanced bacterial clearance in experimental periodontitis models [71,72].

Plasma-activated medium can also activate autophagy via the Akt pathway and is associated with reduced pro-inflammatory macrophage marker expression [69]. CAP-mediated modulation of autophagy therefore represents an important immunomodulatory mechanism that may contribute to bacterial clearance and resolution of inflammation [69,71].

Neutrophil activity

Neutrophils are key components of the host response to periodontal infection; however, excessive neutrophil activation is associated with tissue damage [64,66]. Oliveira et al. reported increased myeloperoxidase (MPO) levels, a biomarker of neutrophil activity, in groups exposed to CAP, suggesting altered neutrophil activity following CAP exposure [73]. Therefore, optimization of CAP treatment parameters may be required to modulate neutrophil activity while limiting adverse tissue effects.

Innate immune signaling pathways

CAP modulates signaling pathways involved in innate immune responses, including pattern recognition receptor (PRR)-mediated inflammatory signaling [59,62,66]. Park et al. reported that CAP suppresses inflammatory responses induced by *P. gingivalis* LPS, a known activator of Toll-like receptor 4 (TLR4) signaling [62]. These findings indicate that CAP influences bacteria-induced innate immune activation. The mechanisms underlying CAP-mediated modulation of PRR signaling remain incompletely understood and may involve effects on receptor expression or downstream signaling molecules. Understanding these mechanisms will be important for optimizing CAP treatment protocols.

Adaptive immune system

CAP can influence the adaptive immune system through effects on T cells and potentially B cell responses and antibody production [59]. Modified CAP treatment has been shown to reduce IL-17 levels in peri-implant sulcular fluid, indicating modulation of Th17-associated inflammatory signaling [64,74]. IL-17, produced mainly by Th17 cells, plays roles in antimicrobial defense and inflammatory tissue responses, but the mechanisms by which CAP affects T cell activation and cytokine profiles remain unclear and require further investigation [64,74].

The effects of CAP on B cell responses and antibody production in periodontitis are not well understood. Since IL-6, which can be reduced by CAP, is involved in B cell differentiation and antibody production, a potential link between CAP exposure and humoral immunity has been suggested [66,67,70]. Further studies are needed to determine whether CAP influences antibody responses to periodontopathogens and how these effects contribute to therapeutic outcomes.

Molecular mechanisms of CAP:

CAP delivers reactive oxygen and nitrogen species (RONS) to tissues, generating secondary RONS in saliva/GCF that mediate cellular responses [31,51,75,76]. These are sensed by redox-sensitive cysteines on signaling proteins (IKK, phosphatases, Keap1, MAPK phosphatases), causing reversible functional changes [59,77]. At therapeutic doses, CAP induces transient oxidative signaling that modulates kinases and promotes regulatory rather than damaging responses [58,59,77,78].

CAP suppresses NF-κB-driven cytokine transcription by oxidizing IKKβ, stabilizing IκBα, and impairing NF-κB DNA binding [78,79]. It also modulates MAPK pathways—reducing p38 and JNK and altering ERK1/2—leading to post-transcriptional cytokine downregulation [80]. CAP further inhibits the NLRP3 inflammasome, decreasing caspase-1 activation and IL-1β maturation [58,81], and promotes macrophage polarization toward an M2 phenotype via inhibition of NF-κB/NLRP3 and activation of Nrf2–HO-1 signaling, lowering TNF-α and IL-1β while increasing IL-10 [58,67,76,77,79].

CAP may also regulate the RANK/RANKL/OPG axis. Studies report reduced RANK/RANKL expression and osteoclast activity after CAP, suggesting attenuation of bone resorption and preservation of alveolar bone [73,82,83,84]. The mechanisms underlying CAP-mediated regulation of the RANK/RANKL/OPG axis remain incompletely defined but likely involve indirect effects on pro-inflammatory cytokines, particularly TNF-α and IL-1β, which are known to upregulate RANKL expression, as well as direct effects on osteoblasts and osteoclasts. Further mechanistic studies are warranted to optimize CAP treatment parameters for maximal bone-preserving effects.

CAP effects on periodontal tissue healing and regeneration:

CAP-treated periodontal fibroblasts transiently upregulate IL-6, IL-8, MCP-1, and GRO-α, supporting leukocyte recruitment and early wound healing, while increasing TGF-β1/2 and PAI-1 expression for tissue remodeling [58,85]. Low CAP-derived H_2_O_2_ acts as a redox signal that activates macrophages, modulates NF-κB, and promotes cytokine release and leukocyte attraction during early healing [58,86,87].

RONS may also modulate PPAR-γ, influencing macrophage polarization and resolution of inflammation through modulation of NF-κB and MAPK pathways, thereby maintaining a controlled pro-healing inflammatory environment [56,73,74,75,78,86,87,88,89,90]. CAP further promotes regeneration by increasing proliferation markers (Ki67, PCNA) and reducing apoptosis markers (Apaf1, p53), supporting cell survival and tissue repair via RONS-mediated regulation of growth-factor and cell-cycle signaling [91,92,93,94].

CAP dose and time dependent effects on cells:

Evidence indicates a dose-dependent therapeutic window for CAP in periodontal therapy [2,92,95]. Short exposures (seconds–minutes) reduce *P. gingivalis* viability while maintaining periodontal cell viability and promoting pro-regenerative gene expression [92,95]. Longer exposures can trigger mild genotoxic or apoptotic effects, with detectable changes reported after ~5–7 min in HGFs and ~3 min in OBA-9 cells [95]. Thus, efficacy depends on careful optimization of plasma source, exposure time, distance, and power to maximize antimicrobial effects while preserving host tissue [2,92,95].

## 4. CAP’s Antibacterial and Immunomodulatory Efficacy

Beyond antimicrobial effects, CAP shows anti-inflammatory and immunomodulatory actions relevant to periodontitis, a host-driven response to dysbiotic biofilm [1,63]. Preclinical and translational evidence indicates CAP can modulate inflammatory signaling, alter cytokine profiles, and support immune-mediated resolution of inflammation [11], positioning it as a promising adjunct targeting both microbial and host components of disease [96].

Antimicrobial and antibiofilm efficacy of CAP

In experimental studies, the use of CAP has been shown to reduce viability in various periodontopathogens [97,98,99]. Multiple studies conducted on the efficacy of CAP against *P. gingivalis* have demonstrated a reduction in viable cells in *P. gingivalis* biofilm following CAP exposure [97,98,99]. The reduction in viability was proportional to CAP exposure time [97,98,99].

A study was performed by Jungbaurer et al. to evaluate CAP’s antibacterial and anti-biofilm effects on 11 oral pathogens including *P. gingivalis*, *Fusobacterium nucleatum*, *Treponema denticola*, and *Tannerella forsythia* [100]. The results demonstrated a significant reduction in all bacterial species after exposure to CAP for 120 s [100]. The following studies demonstrate the antibacterial efficacy of CAP against key periodontal pathogens, suggesting its potential use as an adjunct to conventional periodontal therapy.

Comparative studies evaluating plasma feed gases and device configurations show that helium-based CAP jets (when operating at low discharge power with exposure times between 1 and 7 min) are effective in periodontal biofilm disruption without causing notable genotoxicity [2]. In comparison, argon-based plasmas, including argon supplemented with oxygen, applied for 1 to 5 min, cause substantial biofilm disruption along with synergistic effects when combined with antimicrobial agents [97]. However, there is considerable heterogeneity across studies due to differences in device parameters, including applied voltages and variable pulses.

Immunomodulatory efficacy of CAP

In addition to its antimicrobial properties, CAP exerts pronounced anti-inflammatory effects within periodontal tissues. Experimental studies demonstrate that CAP modulates the expression of key pro-inflammatory cytokines involved in periodontal tissue destruction, including IL-1β, IL-6, IL-8, and TNF-α [62]. These cytokines play central roles in osteoclast activation, matrix metalloproteinase induction, and amplification of inflammatory cascades in periodontitis [62]. Oliveira et al. reported a transient rise in IL-1β after 2 weeks of CAP exposure, followed by a decline at 4 weeks post-exposure [73]. This pattern indicates an early inflammatory response with subsequent resolution, resulting in net anti-inflammatory effects and improved tissue healing. The temporal dynamics of cytokine modulation have implications for treatment scheduling, indicating that repeated CAP application may be required to achieve sustained anti-inflammatory effects.

Eggers et al. found that NIPP decreases IL-1β production in bacteria-stimulated HGFs, suggesting modulation of the inflammatory phenotype of resident periodontal cells [67]. In vivo, Zhou et al. demonstrated that modified CAP lowers IL-1β levels in peri-implant sulcular fluid in a beagle peri-implantitis model, confirming clinically relevant anti-inflammatory effects [74].

Animal models of ligature-induced periodontitis show that CAP application results in significant reduction in pro-inflammatory cytokine levels within periodontal tissues, accompanied by decreased inflammatory cell infiltration and reduced alveolar bone loss [62]. These findings indicate that CAP not only suppresses microbial drivers of inflammation but also directly modulates host immune responses at the tissue level.

Translational studies further report notable reductions in inflammatory mediators within GCF following CAP treatment, suggesting that its immunomodulatory effects extend to clinically measurable biomarkers of periodontal inflammation [63]. When used as an adjunct to SRP, CAP has been associated with reductions in bleeding on probing and gingival inflammation indices, reflecting improved control of the inflammatory burden [4].

At the cellular level, CAP influences immune cell function by enhancing macrophage-mediated clearance of periodontal pathogens through LAP, promoting a shift towards a pro-resolving, anti-inflammatory phenotype [71]. CAP has also been shown to modulate fibroblast and epithelial cell responses, reducing inflammatory signaling while supporting wound healing and tissue repair processes [58,101,102,103].

Collectively, these translational findings support the concept that CAP exerts dual therapeutics effects in periodontology, suppression of pathogenic biofilms and regulation of dysregulated host inflammation. This combined antimicrobial and immunomodulatory action addresses key limitations of current periodontal therapies and underpins the growing interest in CAP as a biologically active adjunctive treatment [11,63,96]. A summary of these key studies is presented in Table 1.

## 5. Effectiveness of CAP in Periodontitis and Comparison with Other Adjuncts

CAP as an adjunct to SRP

A randomized split-mouth clinical trial (*n* = 25) has evaluated NAPP as an adjunct to NSPT in chronic periodontitis [4]. Outcomes at 1 and 3 months included CAL, PPD, PI, GI, BOP, GCF biomarkers, and subgingival microbiology [4]. A single NAPP application produced greater CAL gain in deep pockets (3.9 vs. 3.4 mm), greater bacterial reduction with less recolonization, and significantly lower BOP and GI than NSPT alone [4].

Despite promising results, the small sample size and short (3-month) follow-up limit generalizability and long-term conclusions regarding bone stability and resistance. Larger, long-term trials are needed to confirm safety and reproducibility [4].

Comparing CAP with antibiotics and antiseptics

No clinical or preclinical studies directly comparing CAP with antibiotics or antiseptics for periodontitis were identified. However, one in vitro study evaluated CAP effects on *P. gingivalis* biofilms, using sub-MIC amoxicillin and oxidative agents (hydrogen peroxide, paraquat) to simulate host-derived ROS [97]. CAP reduced biofilm viability, and when combined with sub-MIC amoxicillin during recovery, significantly limited bacterial regrowth compared with amoxicillin alone [97]. CAP-treated biofilms also showed increased susceptibility to oxidative agents, suggesting CAP may sensitize biofilms to ROS-mediated antimicrobial activity [97].

Comparing CAP with antimicrobial photodynamic therapy (aPDT) and laser therapy

Based on our search strategy, no studies were found that compared CAP with aPDT or periodontal laser therapies. This is an important topic with a research gap that provides an opportunity for researchers looking for novel research topics.

## 6. Safety and Biocompatibility Profiles

The clinical use of CAP in periodontology depends on its safety and biocompatibility due to direct tissue contact and RONS generation [104]. In vitro studies show preserved periodontal cell viability under controlled exposure [95], while animal models report no tissue necrosis or harmful inflammation [62,105]. Translational and device studies similarly indicate safe oral application when parameters are properly controlled [96].

Device types

CAP devices used in dental and periodontal research can be broadly classified into plasma jets and dielectric barrier discharge (DBD) systems [103]. Plasma jets generate plasma externally and deliver reactive species to the target area via a gas flow [104]. In contrast, DBD devices generate plasma directly at the tissue surface through electrode–tissue interaction [96]. Both configurations have been explored in periodontal and peri-implant applications [103]. Plasma jets are more frequently investigated for subgingival use due to their localized and non-contact nature, which facilitates access to confined periodontal pockets [103,104].

Technical specifications

The biological effects and safety profile of CAP are strongly influenced by technical parameters such as applied voltage, frequency, power density, gas composition, and the distance between the plasma source and the tissue [103]. Studies included in this review report the use of helium, argon, ambient air, or gas mixtures as carrier gases [103]. Variations in gas composition directly influence the type and concentration of RONS generated during plasma discharge [96]. Optical emission spectroscopy and plasma diagnostics confirm that CAP devices used in periodontal research operate at atmospheric pressure while maintaining low gas temperatures [104]. A summary of the common technical parameters of CAP and their main applications is presented in Table 2.

Clinical applicability and portability of devices

From a clinical perspective, plasma jets offer advantages in accessibility and precision, allowing treatment of periodontal pockets and peri-implant sites without direct tissue contact [104]. Experimental studies demonstrate that plasma jets can be safely extended into periodontal pockets without causing mechanical or thermal tissue damage [104]. Despite these advantages, most CAP systems currently investigated remain laboratory-based or prototype devices [63]. These systems often require trained operators and controlled experimental conditions, limiting routine chair-side use [96]. Translational reviews emphasize that improvements in ergonomics, portability, and reproducibility are required before widespread clinical adoption can be achieved [63].

Cost of devices

The cost of CAP devices varies considerably depending on system complexity, gas delivery requirements, and regulatory compliance [103]. Laboratory-grade plasma systems are typically associated with high initial costs due to specialized power supplies and diagnostic components [96]. These financial requirements may limit accessibility outside academic or research settings [103]. Although simplified and potentially lower-cost systems are under development, economic considerations remain a significant barrier to clinical implementation [63]. The current literature highlights the absence of cost-effectiveness analyses comparing CAP with established adjunctive periodontal therapies [63].

Commercial and laboratory/research devices

At present, the majority of evidence regarding CAP safety and efficacy in periodontology originates from laboratory and experimental devices rather than commercially available dental units [103]. Research systems allow precise control of plasma parameters and exposure conditions [104]. However, these controlled environments may not fully reflect real-world clinical conditions [63]. Differences between laboratory and commercial devices complicate the extrapolation of safety and efficacy data [96]. Standardization of device specifications and validation of commercial systems are therefore essential for successful clinical translation [63].

Thermal measurements

A consistent finding across the included studies is the non-thermal nature of CAP [103]. Thermal measurements conducted during plasma application demonstrate that gas and surface temperatures remain close to room temperature and well below thresholds associated with thermal tissue injury [104]. Rotational temperature analyses confirm that CAP does not induce protein denaturation or coagulative necrosis in periodontal tissues [104]. Animal studies further corroborate these findings by reporting stable tissue temperatures during repeated CAP application [104]. This low thermal output represents a key safety advantage over conventional energy-based dental therapies [96].

Mutagenicity and genotoxicity

The generation of reactive species by CAP raises concerns regarding potential DNA damage [95]. Several studies specifically evaluated genotoxicity in oral and periodontal cells using DNA index analysis and cytometric techniques [95]. These studies demonstrate that CAP exposure at clinically relevant durations does not result in significant genotoxic effects [105]. Mild and transient DNA alterations have been reported at extended exposure times exceeding those required for antimicrobial efficacy [95]. These findings support the existence of a dose-dependent safety window, indicating that genotoxic risk is closely linked to treatment parameters rather than CAP exposure itself [105].

Tissue and cell compatibility

CAP demonstrates a favorable tissue and cell compatibility profile in periodontal-relevant models [95]. In vitro studies show preserved cell viability, normal morphology, and intact cytoskeletal organization of gingival fibroblasts, periodontal ligament cells, and oral keratinocytes following CAP exposure [95]. Importantly, CAP did not induce apoptotic or necrotic changes under therapeutic conditions [70]. Several studies report beneficial biological effects, including enhanced fibroblast adhesion and increased cell migration [70]. Also, CAP has been shown to support wound-healing processes in periodontal tissues [11].

Long-term safety

Despite encouraging short-term safety data, long-term safety evidence remains limited [63]. Animal studies typically involve observation periods of weeks to a few months, during which no adverse local or systemic effects have been reported [104]. Experimental periodontitis models also demonstrate reduced inflammation and bone loss without delayed tissue damage following CAP treatment [62]. Human clinical data remain scarce and are limited to short follow-up durations [63]. Consequently, cumulative exposure effects and delayed adverse outcomes cannot yet be excluded [96].

Contraindications

Based on the available evidence, CAP should be applied with caution in patients with compromised tissue healing or altered immune responses [96]. Conditions associated with impaired oxidative stress regulation may also warrant caution [63]. The safety of CAP in pregnant patients has not been adequately evaluated [96]. Similarly, the use of CAP in medically complex populations should be avoided until further evidence becomes available [63]. Device-specific contraindications include the presence of electronic medical implants and sensitivity to electromagnetic exposure [103].

Recommendations

Based on current evidence, CAP can be considered a safe adjunctive modality in periodontal therapy when used within controlled exposure parameters and with appropriately designed devices [96]. Strict control of treatment parameters is essential to remain within the established therapeutic safety window [105]. Future research should include:

- Standardization of CAP devices and treatment protocols;
- Long-term clinical safety studies with extended follow-up;
- Cost-effectiveness analyses;
- Regulatory validation of commercial dental CAP systems.

Such efforts are essential to support the safe and effective integration of CAP into routine periodontal practice.

## 7. Limitations and Challenges

Standardization and technical challenges

Current studies lack standardized device parameters and treatment protocols, with inconsistent reporting of technical specifications (i.e., voltage, power, frequency, gas composition, and flow rates). Furthermore, there is no consensus on optimal exposure durations or treatment frequencies, making cross-study comparisons difficult and limiting reproducibility. Clinical translation is further constrained by the limited availability of commercially produced, regulatory-approved CAP devices suitable for dental use. Addressing these challenges will require the development of standardized dosimetry frameworks and robust methods for characterizing plasma-generated reactive species to ensure consistency, safety, and therapeutic efficacy in periodontal treatment.

Safety and regulatory concerns

Long-term safety data in human subjects are limited, and current evidence shows an incomplete understanding of genotoxicity thresholds and potential mutagenicity. In addition, the absence of standardized safety guidelines complicates clinical implementation, while uncertainty surrounding regulatory approval pathways for CAP devices further delays translation. To ensure patient safety and sustained clinical efficacy, comprehensive surveillance frameworks are needed to monitor long-term outcomes and identify rare or delayed adverse effects. Safety and regulatory gaps must be addressed before clinical adoption of CAP in periodontology.

Clinical evidence gaps

The available clinical evidence supporting CAP use in periodontology remains limited. Most studies used in vitro cell culture models or experimental animal models of periodontitis which cannot be fully translated to human PD due to the complexity of PD. Furthermore, only a limited number of randomized controlled trials (RCTs) are available, and these involve small sample sizes and insufficient follow-up periods, usually less than three months, thereby restricting assessment of long-term efficacy. Periodontitis is a chronic disease requiring long-term management, and it is crucial to determine whether CAP’s effects are sustained to assess the need for repeated CAP exposures. Moreover, the absence of multicenter studies limits validation across diverse patient populations and clinical settings. Collectively, these factors contribute to insufficient evidence regarding treatment durability, optimal maintenance strategies, and sustainability of clinical outcomes over time.

Research gaps

To use CAP in the management of periodontitis, specifically as an adjunct to conventional periodontal therapy, research gaps must be addressed. There is a knowledge gap in the detailed molecular mechanisms of CAP-generated RONS exposure to specific cellular and tissue responses in PD. For example, researchers are still uncertain about which type of RONS activates which intracellular signaling pathways in periodontal tissues. Additionally, the dose–response relationship between RONS and periodontal cell responses is insufficiently characterized. For example, in PD it is unclear whether chronic low-grade RONS exposure sustains inflammation through maladaptive signaling rather than overt cytotoxicity. Furthermore, RCTs are needed to quantify which patients are expected to benefit most from CAP treatment. Finally, the literature lacks an economic evaluation and cost-effectiveness analyses that compare CAP with other treatments.

## 8. Future Directions and Research Priorities

Despite substantial progress in understanding the antimicrobial, anti-inflammatory, and immunomodulatory potential of CAP in periodontitis, several scientific, technological, and regulatory challenges must be addressed before routine clinical implementation can be achieved. Priority areas for future research include protocol standardization, technology development, robust clinical validation, long-term safety assessment, and exploration of emerging clinical applications [63,96].

Standardization and Clinical Translation

A major barrier to the clinical translation of CAP is the considerable heterogeneity among existing studies. Variations in plasma device type, electrical waveform, discharge power, gas composition and flow rate, temperature, exposure distance, treatment frequency, and application duration directly influence biological outcomes and limit meaningful comparison across investigations [104]. The establishment of standardized reporting frameworks incorporating plasma dosimetry, electrical parameters, gas characteristics, thermal measurements, and exposure geometry would improve reproducibility and facilitate data synthesis. Similarly, the adoption of standardized clinical outcome measures, including probing pocket depth (PPD), clinical attachment level (CAL), bleeding on probing (BOP), microbial recolonization patterns, and gingival crevicular fluid (GCF) biomarkers, would strengthen evidence generation and support future meta-analyses.

Technology Development

Continued technological innovation is essential to improve the practicality, safety, and therapeutic performance of CAP systems. Future developments should focus on device miniaturization, improved handpiece ergonomics, integrated temperature monitoring, and dose-control mechanisms to ensure safe and reproducible clinical application. Advances in portable gas delivery systems, such as compact gas cartridges, may eliminate reliance on external gas cylinders and facilitate routine use in dental practice. Furthermore, the development of multifunctional platforms that combine CAP with complementary therapeutic modalities could expand its clinical utility and enhance treatment efficacy.

Clinical Validation and Personalized Treatment Approaches

The current clinical evidence base remains limited by small sample sizes, short follow-up periods, and predominantly single-center study designs. Large-scale, adequately powered, multicenter randomized controlled trials with extended follow-up periods are required to establish both short- and long-term clinical effectiveness. Inclusion of patient-reported outcomes, treatment durability, and cost-effectiveness analyses would further enhance the clinical relevance of future investigations. In addition to mechanistic studies and randomized clinical trials, future research should evaluate the economic feasibility of CAP-based periodontal therapies. Cost-effectiveness and cost–benefit analyses are required to determine whether the clinical benefits of CAP justify the costs associated with plasma-generation devices, maintenance, training, and implementation in routine dental practice. Such data will be essential for facilitating evidence-based adoption and regulatory approval of CAP technologies.

As understanding of CAP–host interactions continues to evolve, opportunities for personalized treatment strategies may emerge. Future research should explore the development of personalized CAP treatment protocols tailored to individual patient characteristics. Parameters such as plasma dose, exposure time, treatment frequency, and gas composition may require adjustment based on periodontal pocket depth, microbial composition, disease severity, and local inflammatory status. For example, sites exhibiting deeper periodontal pockets or higher levels of pathogenic anaerobic bacteria may benefit from longer exposure times or repeated CAP applications, whereas variations in gas composition may be optimized to enhance antimicrobial activity against specific microbial communities while minimizing effects on surrounding tissues. The integration of microbiological, clinical, and host-response data may ultimately facilitate the development of patient-specific CAP treatment strategies for precision periodontal therapy.

Combination approaches also warrant further investigation. Integrating CAP with conventional periodontal therapy, antimicrobial photodynamic therapy, regenerative procedures, or biomaterial-based interventions may produce synergistic effects through simultaneous modulation of microbial, inflammatory, and regenerative pathways.

Long-Term Safety and Regulatory Considerations

Although current studies generally support the safety and biocompatibility of CAP, future research should include longitudinal animal studies and clinical investigations with extended follow-up periods to evaluate the long-term biological effects of repeated CAP exposure. Particular attention should be given to the assessment of cumulative oxidative stress, delayed tissue responses, genomic stability, and potential genotoxic effects associated with chronic or repeated treatments. Such studies will be essential for establishing long-term safety profiles, defining safe exposure thresholds, and facilitating the clinical translation of CAP-based periodontal therapies. In parallel, successful clinical translation will require collaboration with regulatory agencies to establish clear approval pathways for plasma-based medical devices. Demonstration of device reliability, manufacturing consistency, clinical efficacy, and long-term safety will be essential for regulatory approval. Following market introduction, post-market surveillance systems should be implemented to monitor long-term treatment outcomes and identify rare adverse events.

Emerging Applications

The potential applications of CAP in periodontics extend beyond its use as an adjunct to conventional non-surgical periodontal therapy [11]. Emerging evidence suggests a potential role in the management of peri-implant diseases through simultaneous reduction in microbial burden and modulation of peri-implant inflammation [11]. CAP may also support periodontal regeneration by enhancing the outcomes of guided tissue regeneration procedures and improving the biological performance of regenerative biomaterials [11]. Integration of CAP with scaffolds, growth factors, and other regenerative technologies represents a promising area of future investigation. Additional applications may include pre-surgical site decontamination and maintenance-phase periodontal therapy aimed at improving long-term disease control in susceptible patients [11]. Continued advances in device technology and clinical evidence generation will help define the full therapeutic potential of CAP within contemporary periodontal practice.

## 9. Discussion

The evidence reviewed in this manuscript collectively supports the biological plausibility of CAP as an adjunctive modality in the management of periodontitis. Across in vitro and preclinical models, CAP consistently demonstrates potent antimicrobial activity against key periodontal pathogens, effective disruption of mature biofilms, and meaningful modulation of the host inflammatory response through RONS-mediated signaling [11,63,96]. Importantly, these effects operate through multiple simultaneous mechanisms—targeting bacterial membranes, intracellular components, and immune signaling pathways—which distinguishes CAP from conventional single-target adjuncts and reduces the likelihood of resistance development [13,20]. When used alongside SRP, preliminary clinical evidence suggests improvements in CAL, reductions in BOP, and decreased recolonization by periodontal pathogens, indicating that the biological activity observed preclinically does translate, at least in part, to the clinical setting [4].

However, the transition from promising preclinical findings to established clinical evidence remains incomplete. The available clinical data are largely derived from a small number of trials with limited sample sizes and follow-up periods not exceeding three months, which is a significant limitation given the chronic and relapsing nature of periodontitis [4]. Long-term outcomes, including the durability of CAL gains, sustained bacterial suppression, and the need for repeated CAP exposures during maintenance therapy, have not been adequately characterized [63]. Furthermore, most studies have been conducted in controlled experimental settings using laboratory-grade devices, which may not reflect the conditions and patient variability encountered in routine clinical practice [63,96]. These gaps mean that, at present, CAP should be regarded as a promising investigational adjunct rather than an intervention ready for widespread clinical adoption [63].

A further challenge is the considerable heterogeneity in CAP systems and treatment protocols, with variations in device type, carrier gas, exposure duration, and delivery method. These variations may influence the generation of reactive species making cross-study comparisons difficult [104]. Therefore, direct comparison of findings across studies should be interpreted with caution. It is worth noting that restricting this review to a single CAP-generation system, while methodologically appealing, would have excluded the majority of the available literature and obscured the broader mechanistic picture that emerges when different plasma configurations are considered together. As discussed in Section 7, the absence of standardized reporting frameworks compounds this issue, and establishing consensus on plasma dosimetry and outcome reporting remains a prerequisite for building a reliable evidence base [104]. Notably, direct comparisons with established adjuncts such as locally delivered antimicrobials and aPDT are currently absent, which limits the ability to position CAP within the existing treatment hierarchy and identify the clinical scenarios in which it may offer the greatest benefit.

## 10. Conclusions

This literature review highlights CAP as a promising adjunctive agent in the management of periodontitis. Accumulating experimental and preclinical evidence shows that CAP exerts potent antimicrobial effects against key periodontal pathogens, disrupts mature biofilms through RONS-mediated mechanisms, and enhances the susceptibility of residual biofilms to conventional antimicrobial agents. Beyond its antimicrobial activity, CAP modulates inflammatory responses by reducing pro-inflammatory cytokine expression and suppressing osteoclast-mediated bone resorption. Those effects appear to be context-dependent, supporting a balanced immunomodulatory profile that promotes resolution of inflammation while preserving tissue repair mechanisms. Furthermore, CAP influences the innate and adaptive immune systems, enhances macrophage bactericidal function, and modulates immune cell polarization, thus promoting host-microbiome homeostasis. In parallel, CAP shows regenerative potential in periodontics by promoting PDLCs proliferation, migration, wound healing, increasing the expression of ECM and regenerative markers, and accelerating tissue recovery in preclinical models.

Although CAP exhibits promising antimicrobial, immunomodulatory, and regenerative properties, current clinical evidence remains limited and is substantially outweighed by preclinical data. Therefore, CAP should presently be considered a promising adjunctive approach rather than an established standard of care for periodontitis. Further large-scale robust RCTs, safety monitoring, and long-term clinical studies are required before definitive recommendations for routine clinical implementation can be made. Additionally, further mechanistic studies in human periodontal tissues are needed to demonstrate efficacy, establish dosing regimens, and offer guidance on clinical indications.

The evidence on the efficacy of CAP in the management of periodontitis is accumulatively increasing, suggesting its potential use as an adjunct to SRP in the future due to its unique antimicrobial, immunomodulatory, and regenerative properties. Utilizing this potential relies on the continuation of rigorous research, technological advancements, and clinical validation, which could provide dental practitioners with a new option for the management of PD.

## Figures and Tables

**Table 1:** Summary of key studies evaluating the antibacterial and immunomodulatory efficacy of cold atmospheric plasma (CAP) on periodontitis.

Study	Model/Design	CAP Type & Key Parameters	Key Findings
Jungbauer et al., 2022 [100]	In vitro; 11 oral bacteria and 12-species biofilms on dentin and titanium disks	Handheld piezoelectric direct-discharge device; ambient air; 10–60–120 s	≥3 log_10_ CFU reduction in 10/11 species at 30 s (*T. forsythia* required 60 s); all species at 60 s. Biofilm metabolic activity reduced 95–98% at 120 s; no cytotoxicity to gingival fibroblasts.
Hong et al., 2022 [97]	In vitro; *P. gingivalis* biofilms on stainless-steel coupons	DC-driven non-thermal plasma “brush”; pure argon and argon + oxygen; 1, 2, 5 min	Up to 2.7 log_10_ reduction in viable load, proportional to exposure time. Synergy with sub-MIC antibiotics and oxidative stress; impaired biofilm re-colonization.
Eggers et al., 2023 [67]	In vitro; human gingival fibroblasts and keratinocytes	kINPen MED non-invasive physical plasma jet; argon at 4.0 SLM; 30–60–90 s (30 s optimal)	No loss of cell viability. Downregulation of pro-inflammatory mediators including IL-1β, IL-6, IL-8 and TNF at the protein level, supporting an anti-inflammatory effect.
Lima et al., 2021 [3]	In vitro (mono- and dual-species biofilm; HGFs) and in vivo (mouse ligature-induced periodontitis)	Low-power helium CAP jet; 1–3–5–7 min (in vitro); 5 min/session (in vivo)	Significant biofilm reduction from 3 min upward; no notable cytotoxicity/genotoxicity to fibroblasts. In vivo, repeated CAP showed a trend toward improved bone architecture and collagen recovery.
Yu et al., 2025 [71]	In vitro (macrophages with *P. gingivalis*) and in vivo (mouse experimental periodontitis)	Handheld helium CAP jet; bipolar pulsed −5 kV/+10 kV; 80 s (in vitro), 120 s (in vivo)	CAP alleviated experimental periodontitis and enhanced macrophage intracellular killing of *P. gingivalis* in a ROS-dependent manner via LC3-associated phagocytosis (LAP).
Oliveira et al., 2024 [73]	In vivo; rat experimental periodontitis (2- and 4-week endpoints)	Atmospheric (cold) plasma; device gas, exposure and power not specified	IL-1β rose at 2 weeks then declined at 4 weeks; TNF-α lower and IL-10 higher vs controls at 4 weeks. Reduced inflammatory infiltrate; RANK and RANKL decreased.
Zhou et al., 2022 [74]	In vivo; beagle ligature-induced peri-implantitis	Modified cold atmospheric plasma (plasma + chlorhexidine); device parameters not specified	Adjunctive MCAP improved sulcus bleeding index, probing depth and bone height vs chlorhexidine alone. IL-1β and IL-17 significantly decreased in peri-implant sulcular fluid; IL-6 unchanged.
Zhang et al., 2018 [84]	In vivo; rat ligature-induced periodontitis; adjunct to SRP (14- and 30-day endpoints)	DC-driven non-thermal atmospheric plasma; adjunct to scaling and root planing	SRP + plasma reduced alveolar bone loss vs SRP alone (BV/TV increased ~40% at 30 days), with fewer osteoclasts and lower RANKL. TNF-α and IL-1β decreased; IL-10 increased.

CFU, colony-forming units; HGF, human gingival fibroblast; SRP, scaling and root planing; RANKL, receptor activator of nuclear factor-κB ligand; SLM, standard liters per minute; LAP, LC3-associated phagocytosis.

**Table 2:** Principal Technical Parameters of CAP Systems Evaluated in Periodontal Research.

Study	Device Type	Gas Composition	Exposure Time	Electrical Parameters	Main Application
Rupf et al., 2010 [57]	Plasma jet	Helium	1–7 min	Low discharge power	Antimicrobial activity and biofilm reduction with minimal genotoxic effects
Jungbauer et al., 2022 [100]	Plasma jet	Ambient air	120 s	Atmospheric-pressure operation	Broad-spectrum antimicrobial activity against periodontal pathogens
Eggers et al., 2023 [67]	Non-invasive physical plasma (NIPP)	Argon	Variable	Device-specific parameters	Reduction in inflammatory cytokine production in human gingival fibroblasts
Oliveira et al., 2024 [73]	CAP device (animal model)	Not specified	Repeated applications	Atmospheric-pressure plasma	Modulation of inflammatory mediators and preservation of alveolar bone
Zhou et al., 2022 [74]	Modified CAP system	Not specified	Experimental application	Atmospheric-pressure plasma	Reduction in peri-implant inflammatory biomarkers
Küçük et al., 2020 [4]	Non-thermal atmospheric-pressure plasma (NAPP)	Helium-based plasma jet	Single application	Low-temperature plasma discharge	Adjunctive periodontal therapy with improved clinical and microbiological outcomes

CAP, cold atmospheric plasma; NAPP, non-thermal atmospheric-pressure plasma; NIPP, non-invasive physical plasma.

## Data Availability

No new data were created or analyzed in this study. Data sharing is not applicable to this article.

## Abbreviations

The following abbreviations are used in this manuscript:

Apaf1	Apoptotic protease activating factor 1
aPDT	Antimicrobial photodynamic therapy
BOP	Bleeding on probing
CAL	Clinical attachment level
CAP	Cold atmospheric plasma
CXCL8	C-X-C motif chemokine ligand 8
DBD	Dielectric barrier discharge
DNA	Deoxyribonucleic acid
ECM	Extracellular matrix
eDNA	Extracellular DNA
ERK	Extracellular signal-regulated kinase
EPS	Extracellular polymeric substance
GCF	Gingival crevicular fluid
GI	Gingival index
GRO-α	Growth-related oncogene alpha
H_2_O_2_	Hydrogen peroxide
HGF	Human gingival fibroblast
HO-1	Heme oxygenase-1
IKK	IκB kinase
IκB	Inhibitor of nuclear factor kappa B
IL	Interleukin
JNK	c-Jun N-terminal kinase
Keap1	Kelch-like ECH-associated protein 1
Ki67	Marker of proliferation Ki-67
LAP	LC3-associated phagocytosis
LC3	Microtubule-associated protein 1A/1B-light chain 3
LPS	Lipopolysaccharide
MAPK	Mitogen-activated protein kinase
MCP-1	Monocyte chemoattractant protein-1
MIC	Minimum inhibitory concentration
MMP	Matrix metalloproteinase
MPO	Myeloperoxidase
mRNA	Messenger ribonucleic acid
NAPP	Non-thermal atmospheric pressure plasma
NF-κB	Nuclear factor kappa B
NIPP	Non-invasive physical plasma
NLRP3	NOD-like receptor family pyrin domain containing 3
NO	Nitric oxide
NSPT	Non-surgical periodontal therapy
O_3_	Ozone
OBA-9	Oral epithelial cell line
OPG	Osteoprotegerin
PAI-1	Plasminogen activator inhibitor-1
PAM	Plasma-activated medium
PCNA	Proliferating cell nuclear antigen
PD	Periodontal disease
PDLC	Periodontal ligament cell
PI	Plaque index
PPAR-γ	Peroxisome proliferator-activated receptor gamma
PPD	Probing pocket depth
PRR	Pattern recognition receptor
QS	Quorum sensing
RANK	Receptor activator of nuclear factor kappa B
RANKL	Receptor activator of nuclear factor kappa B ligand
RNS	Reactive nitrogen species
RONS	Reactive oxygen and nitrogen species
ROS	Reactive oxygen species
SRP	Scaling and root planing
TGF-β	Transforming growth factor beta
Th17	T helper 17
TLR4	Toll-like receptor 4
TNF-α	Tumor necrosis factor alpha
UVA	Ultraviolet A
UVB	Ultraviolet B
UVC	Ultraviolet C

## Appendix A

**Table A1:** Key Periodontal Cytokines and CAP Effects.

Cytokine	Role in Periodontitis	Effect of CAP	Mechanistic Basis
IL-1β	Bone resorption, MMP activation	↓ Downregulated	NF-κB inhibition, reduced inflammasome activation
TNF-α	Osteoclastogenesis, tissue breakdown	↓ Downregulated	Suppressed MAPK and NF-κB signaling
IL-6	Chronic inflammation, Th17 activation	↓ Dose-dependent reduction	Suppressed MAPK and NF-κB signaling
IL-8 (CXCL8)	Neutrophil recruitment	↓ Reduced	Attenuated epithelial stress signaling
IL-17	Bone loss amplification	↓ Indirect reduction	Not fully understood
RANKL	Osteoclast differentiation	↓ Reduced	TNF-α and IL-1β suppression
OPG	Bone protection	↑ Relative increase	RANKL downregulation
IL-10	Anti-inflammatory	↑ Upregulated	M2 macrophage polarization
TGF-β	Tissue repair, fibrosis control	↑ Context-dependent increase	Fibroblast redox signaling

Summary of key cytokines involved in the pathogenesis of periodontitis and their modulation by cold atmospheric plasma (CAP). Pro-inflammatory and bone-resorptive mediators, including IL-1β, TNF-α, IL-6, IL-8 (CXCL8), IL-17, and RANKL, are generally downregulated following CAP exposure, reflecting suppression of inflammatory signaling pathways such as NF-κB, and MAPK. In contrast, anti-inflammatory and tissue-protective mediators, including IL-10, osteoprotegerin (OPG), and transforming growth factor-β (TGF-β), show relative upregulation or context-dependent increases, consistent with CAP-induced redox modulation, macrophage polarization, and promotion of regenerative responses.
